# Blocking the Nav1.8 channel in the left stellate ganglion suppresses ventricular arrhythmia induced by acute ischemia in a canine model

**DOI:** 10.1038/s41598-017-00642-6

**Published:** 2017-04-03

**Authors:** Lilei Yu, Menglong Wang, Dan Hu, Bing Huang, Liping Zhou, Xiaoya Zhou, Zhuo Wang, Songyun Wang, Hong Jiang

**Affiliations:** 1 Department of Cardiology, Renmin Hospital of Wuhan University, Cardiovascular Research Institute, Wuhan University, Hubei Key Laboratory of Cardiology, Wuhan, China; 2 Masonic Medical Research Laboratory, 2150 Bleecker Street, Utica, New York 13501-1787 USA

## Abstract

Left stellate ganglion (LSG) hyperactivity promotes ischemia induced ventricular arrhythmia (VA). Blocking the Nav1.8 channel decreases neuron activity. Therefore, the present study aimed to investigate whether blocking the Nav1.8 channel with its specific blocker A-803467 in the LSG reduces sympathetic activity and exerts anti-arrhythmic effects. Forty canines were divided into dimethylsulfoxide (DMSO) group and 10 mM, 15 mM, and 20 mM A-803467 groups. A volume of 0.1 ml of A-803467 or DMSO was injected into the LSG. The ventricular electrophysiological parameters, LSG function were measured before and 30 min after the injection. VA was assessed for 60 min after ischemia and then LSG tissues were collected for molecular biological experiments. Compared with DMSO, concentration-dependent prolonged action potential duration and effective refractory period, decreased LSG function were identified after A-803467 treatment. Moreover, the severity of ischemia induced VA was decreased in A-803467 groups. Furthermore, decreased nerve growth factor, decreased c-fos and increased sympathetic neuron apoptosis were found in the LSG after A-803467 injection. In conclusion, blocking the Nav1.8 channel could significantly attenuate ischemia-induced VA, primarily by suppressing LSG activity.

## Introduction

Ventricular arrhythmia (VA) is a major cause of cardiac death after myocardial infarction. Recently, studies have demonstrated that the left stellate ganglion (LSG) is the key factor that contributes to initializing and maintaining VA^1–3^. Direct nerve activity recording has shown that LSG neural activity is significantly increased before VA onset^4^. Furthermore, direct infusion of nerve growth factor (NGF) into the LSG or sub-threshold electric stimulation of the LSG increases the incidence rate of ventricular fibrillation (VF) and sudden cardiac death^5, 6^. Both clinical and basic studies have revealed that LSG denervation prevents the recurrence of malignant VA^7, 8^. However, the clinical use of direct LSG resection is limited due to its side effects, such as Horner’s syndrome and accidental hemorrhages^9, 10^. New methods targeting LSG denervation are promising.

The Nav1.8 channel, a voltage-gated sodium channel encoded by SCN10A and mainly located in afferent neurons, was first discovered in dorsal root ganglion (DRG) neurons^11, 12^. Studies have demonstrated that the Nav1.8 channel plays an important role in peripheral pain processing^13, 14^. Selective pharmacological blockade of the Nav1.8 channel in the DRG exerts a significant antinociception function by inhibiting neural activity^15, 16^. More importantly, blocking the Nav1.8 channel with its specific blocker A-803467 (5-[4-Chlorophenyl]-N-[3, 5-dimethoxyphenyl] furan-2-carboxamide]) in the cardiac ganglionated plexi (GP) decreases atrial fibrillation inducibility^17, 18^. All these results indicate that the Nav1.8 channel plays important roles in regulating neural activity and suggest a potential role of the Nav1.8 channel in the LSG. In the present study, we aimed to investigate whether blocking the Nav1.8 channel with its specific blocker A-803467 in the LSG can decrease the incidence of VA in an acute ischemia canine model.

## Results

**Figure 1:**
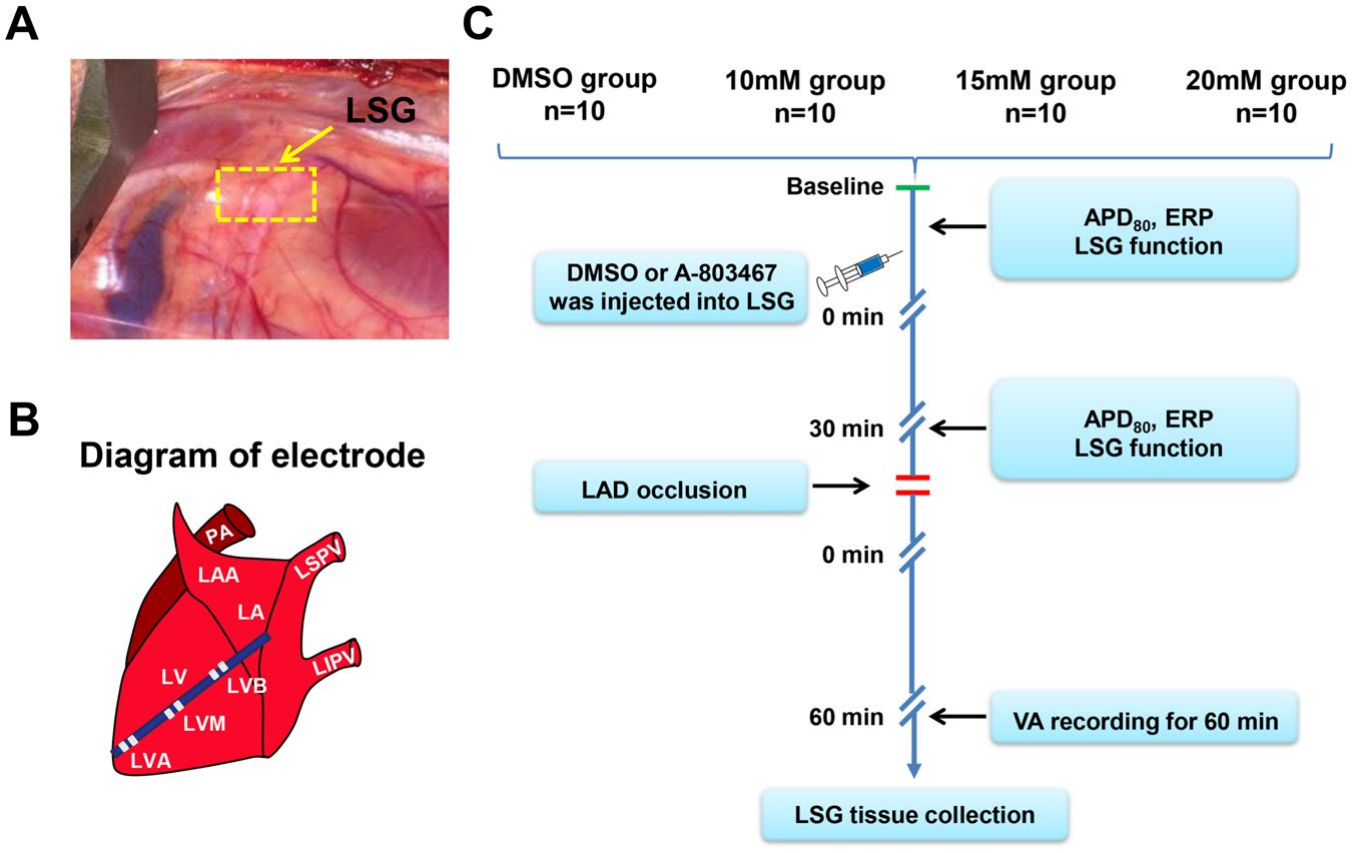
The LSG location (**A**), schematic representation of electrodes position in the left ventricular free walls (**B**) and the experimental design flowchart were shown (**C**). LSG = left stellate ganglion; LAA = left atrial appendage; LA = left atria; PA = pulmonary artery; LSPV = left superior pulmonary vein; LIPV = left inferior pulmonary vein; LV = left ventricular; LVA = left ventricular apex; LVB = left ventricular base; LVM = median area between LVA and LVB; DMSO = dimethylsulfoxide; APD = action potential duration; ERP = effective refractory period; LAD = left anterior descending coronary artery; VA = ventricular arrhythmia.

The LSG was exposed (Fig. 1A), and three electrodes were attached at three epicardial sites: the left ventricular apex (LVA), the left ventricular base (LVB) and the median area between the LVA and the LVB (LVM), as shown in the schematic diagram (Fig. 1B). The experiment was completed in accordance with the flow chart (Fig. 1C). All canines developed acute ST-segment and T-wave changes on surface ECGs after ligation of the left anterior descending artery (LAD).

### Effect of blocking Nav1.8 channel on the ventricular APD_80_ and ERP in normal hearts

**Figure 2:**
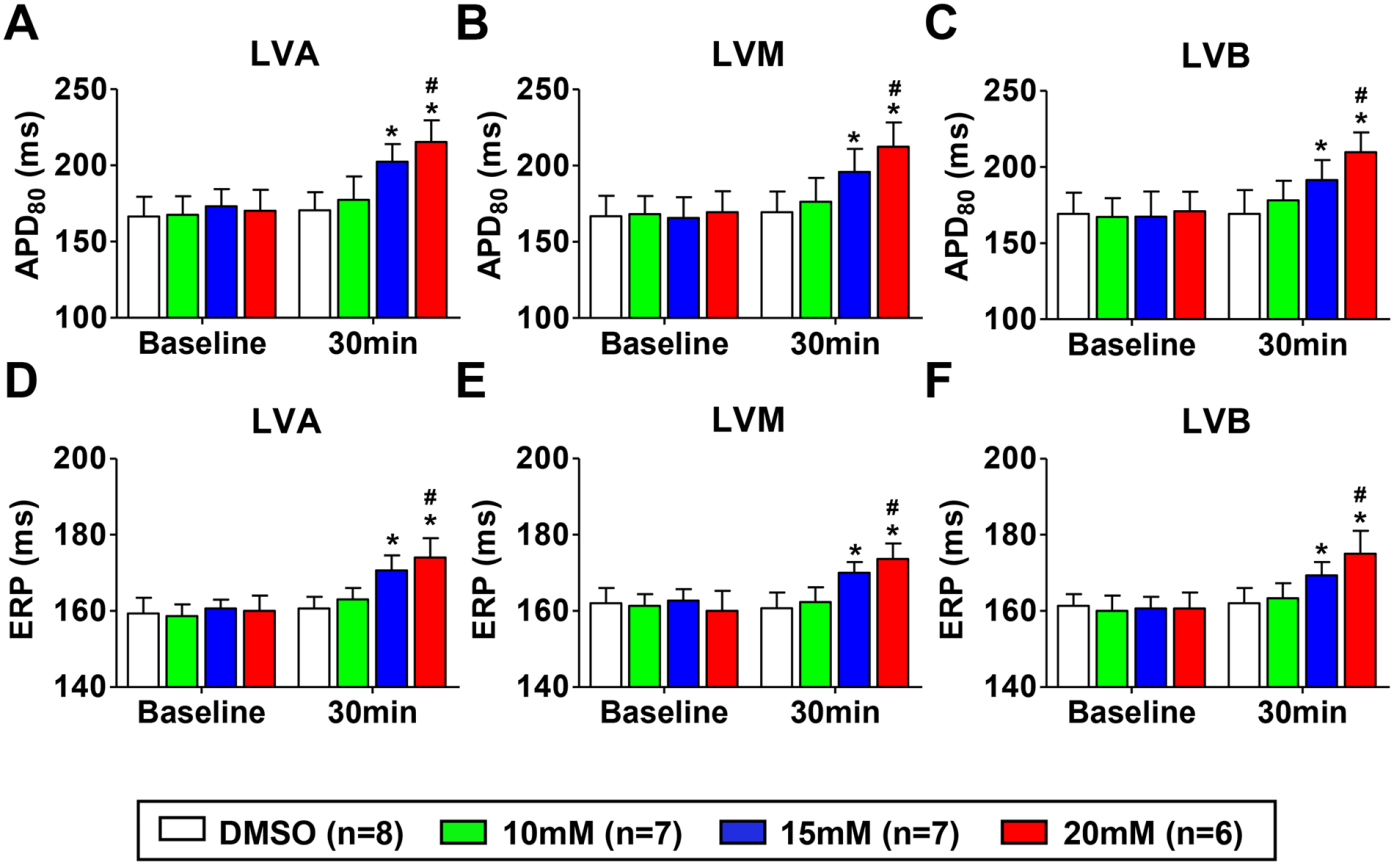
Effects of Nav1.8 channel block on the ventricular APD_80_ (**A–C**) and ERP (**D–F**). Blocking Nav1.8 channel with A-803467 significantly prolonged ventricular APD_80_ and ERP, whereas no significantly change was found in the DMSO group. *p < 0.05 vs DMSO group at 30 min, ^#^p < 0.05 vs 10 mM group at 30 min. The abbreviations were similar with Fig. 1.

The left ventricular action potential duration (APD) and effective refractory period (ERP) were assessed at three epicardial sites (LVA, LVM and LVB) in all canines and the examples of original traces for APD and ERP were presented as Supplementary Fig. S1. No difference in the APD_80_ or ERP was observed between the dimethylsulfoxide (DMSO) group and the A-803467 treatment groups at baseline. However, a concentration-dependent prolonged APD_80_ (Fig. 2A–C) and ERP (Fig. 2D–F) were identified in the A-803467 treatment groups compared with the DMSO group. For example, the APD_80_ at the LVA after A-803467 treatment was 170.6 ± 11.9 ms, 177.4 ± 15.4 ms, 202.4 ± 11.7 ms and 215.4 ± 14.2 ms for the DMSO group and the 10 mM, 15 mM and 20 mM A-803467 groups, respectively.

### Effect of blocking Nav1.8 channel on VA in ischemic hearts

**Figure 3:**
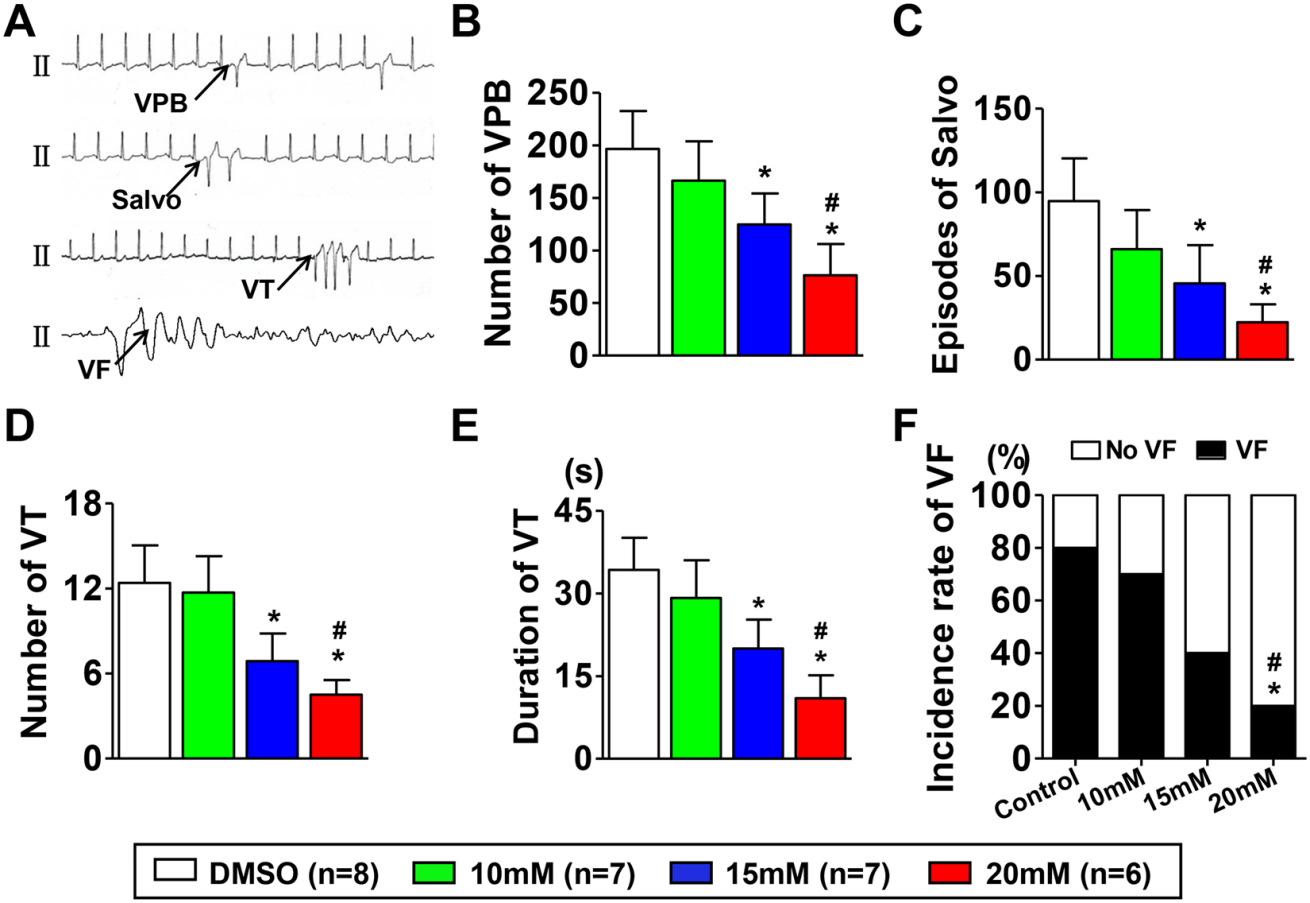
Effects of Nav1.8 channel block on VA episodes. (**A**) Representative ECG examples. (**B–E**) Quantitative analysis of the number and duration of different types of VA. *p < 0.05 vs DMSO group, ^#^p < 0.05 vs 10 mM group. VA = ventricular arrhythmia; VPB = ventricular premature beat; VT = ventricular tachycardia; VF = ventricular fibrillation.

VAs were induced by acute ischemia, as demonstrated by the occurrence of ventricular premature beats (VPBs), salvo beats, ventricular tachycardia (VT), and VF in the DMSO group during the 60 min continuous electrocardiograph (ECG) recording after LAD occlusion (Fig. 3A). However, the number of VPBs and salvo beats in the A-803467 treatment groups was significantly decreased compared with the DMSO group (Fig. 3B and C). In addition, the number of VT episodes was significantly decreased in the A-803467 treatment groups (Fig. 3D). Furthermore, the average VT duration in the A-803467 treatment groups was significantly shorter than that in the DMSO group (34.3 ± 5.8 ms for the DMSO group and 11.0 ± 4.1 ms for the 20 mM group) (Fig. 3E). Finally, the incidence rate of VF was significantly lower in the 20 mM A-803467 treatment group than that in DMSO group and 10 mM A-803467 group (Fig. 3F). These results indicated that blocking Nav1.8 channel significantly attenuated the severity of VA induced by ischemia.

### Effect of blocking Nav1.8 channel on LSG activity

**Figure 4:**
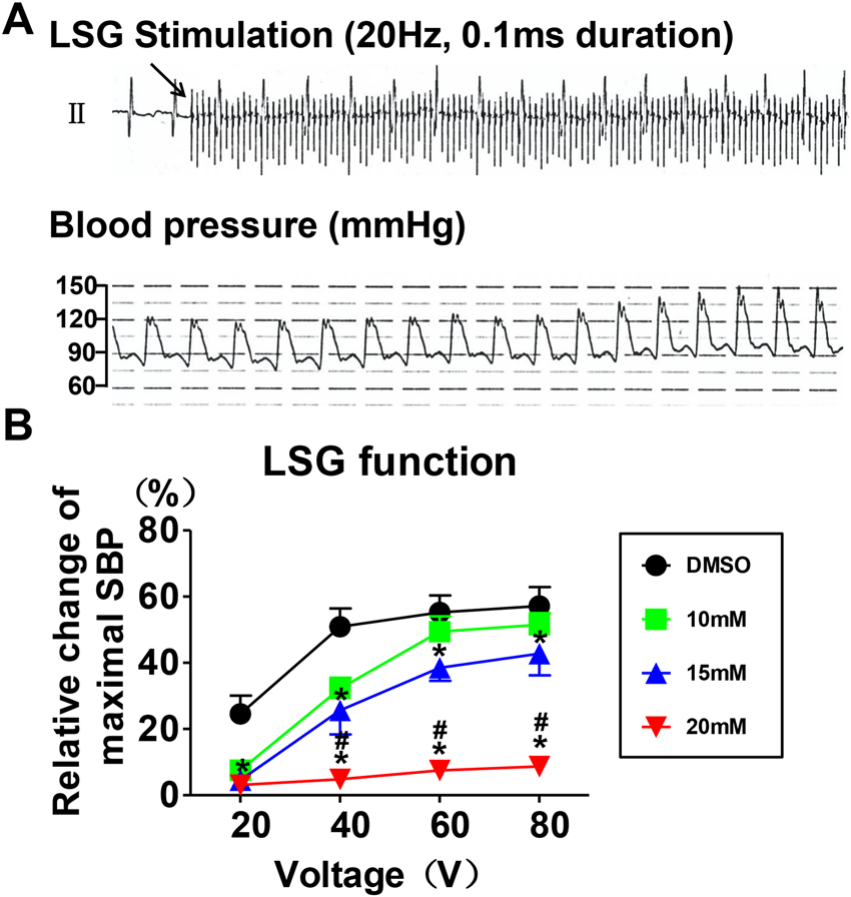
Effects of Nav1.8 channel block on LSG function. (**A**) The representative BP elevation example in response to LSG electrical stimulation. (**B**) The voltage/BP response curve in different groups. Blocking Nav1.8 channel attenuated LSG function. *p < 0.05 vs DMSO group, ^#^p < 0.05 vs 10 mM group. SBP = systolic blood pressure.

The LSG function, which was measured by the systolic blood pressure (SBP) elevation response after LSG electrical stimulation, was used to indicate LSG activity. Figure 4A displays an example of the SBP elevation in response to LSG electrical stimulation. The results showed that blocking the Nav1.8 channel blunted the LSG function, as demonstrated by a decreased change in the maximal SBP at the same voltage level of electrical stimulation (Fig. 4B). Notably, it appeared that treatment with a high concentration of A-803467 (20 mM) almost suppressed the LSG function.

### Effect of blocking Nav1.8 channel on the expression of NGF and c-fos in the LSG

**Figure 5:**
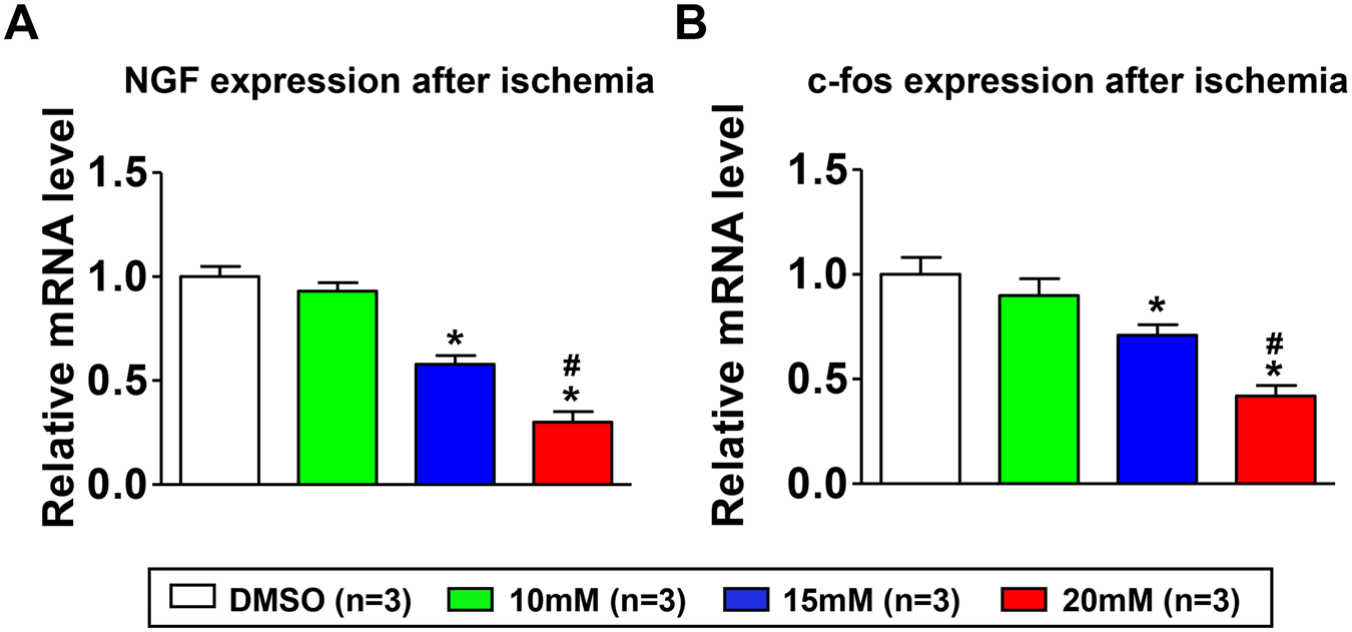
Effect of Nav1.8 channel block on the mRNA expression of NGF (**A**) and c-fos (**B**) in LSG. NGF = nerve growth factor. *p < 0.05 vs DMSO group, ^#^p < 0.05 vs 10 mM group.

The expression of c-fos was used as a rapid indicator for extensive activation of neural elements^19^, meanwhile NGF expression was an important contributor to the pathological remodeling of LSG in response to ischemia^20^. Firstly, the levels of NGF in the LSG collected from the DMSO group and from normal canines without any intervention were compared. The results showed that 60 min ischemia did not increase the mRNA expression of NGF (see Supplementary Fig. S2). Then, the mRNA expression of NGF in the DMSO group and A-803467 groups was evaluated. As shown in Fig. 5A, blocking the Nav1.8 channel in the LSG significantly reduced mRNA levels of NGF and this inhibitory effect on sympathetic remodeling was enhanced at higher A-803467 concentration. In addition, the expression of c-fos in the DMSO group and the A-803467 groups showed the same tendency as NGF expression (Fig. 5B).

### Effect of blocking Nav1.8 channel on the apoptosis of TH-positive neurons in the LSG

**Figure 6:**
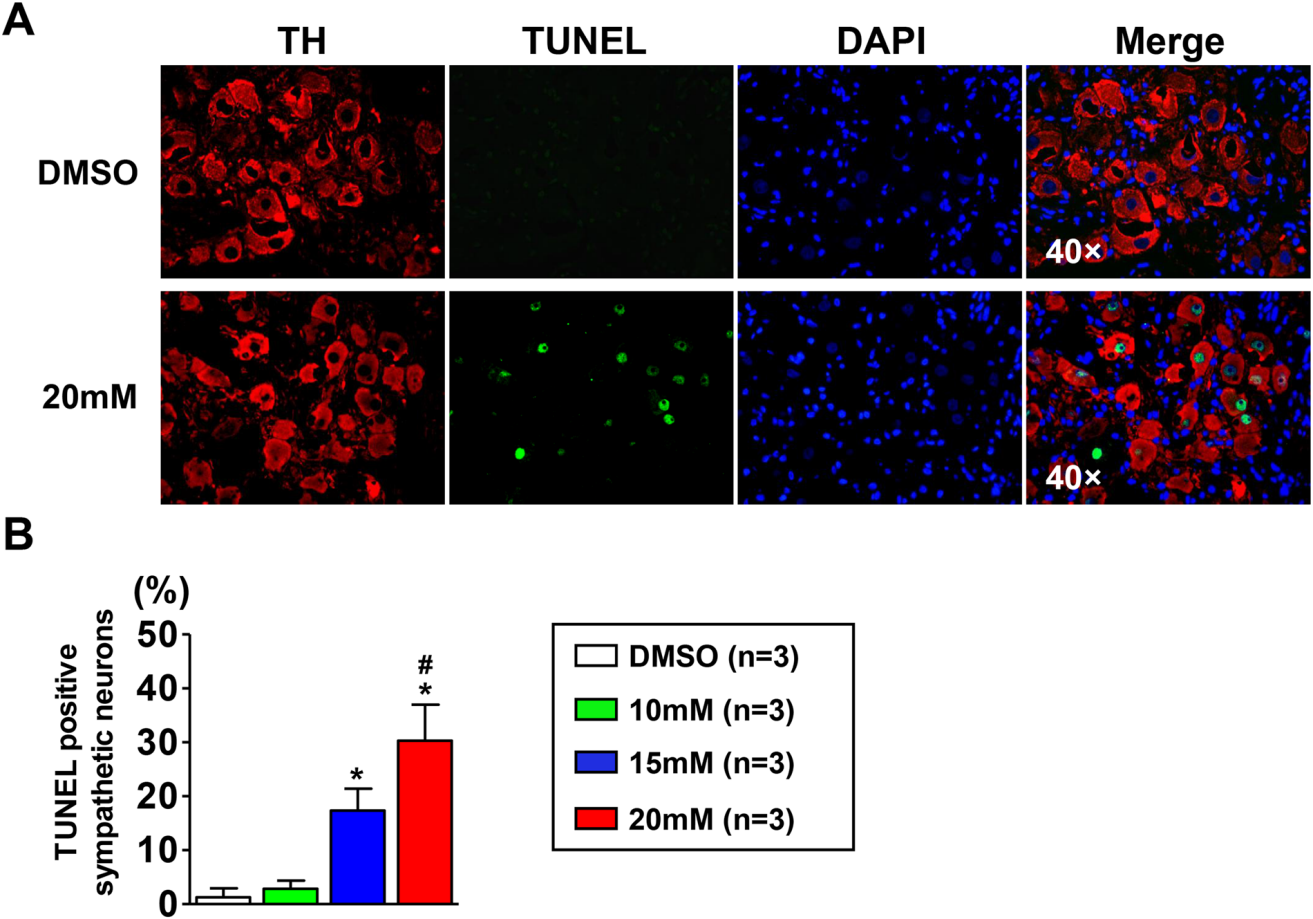
Effect of Nav1.8 channel block on apoptosis of sympathetic neurons in LSG. (**A**) Representative images showing double-immunofluorescence staining for TH (red) and TUNEL (green) in LSG from DMSO group and 20 mM A-803467 group. (**B**) Quantification of the sympathetic neuron apoptosis (n = 3, *p < 0.05 vs DMSO group, ^#^p < 0.05 vs 10 mM group). TH = Tyrosine hydroxylase.

Double staining of tyrosine hydroxylase (TH) and terminal-deoxynucleoitidyl Transferase Mediated Nick End Labeling (TUNEL) was applied to detect the severity of sympathetic neuron apoptosis in the LSG. As shown in Fig. 6A, the ganglion neurons in the DMSO group were mostly stained positive for TH (red) and negative for TUNEL (green). However, in the high-concentration A-803467 treatment group, most ganglion neurons stained positive for both TH and TUNEL. The quantitative analysis showed that the apoptosis rate was higher in the high concentration group than that in the DMSO and low-concentration groups (Fig. 6B).

## Discussion

### Major findings

**Figure 7:**
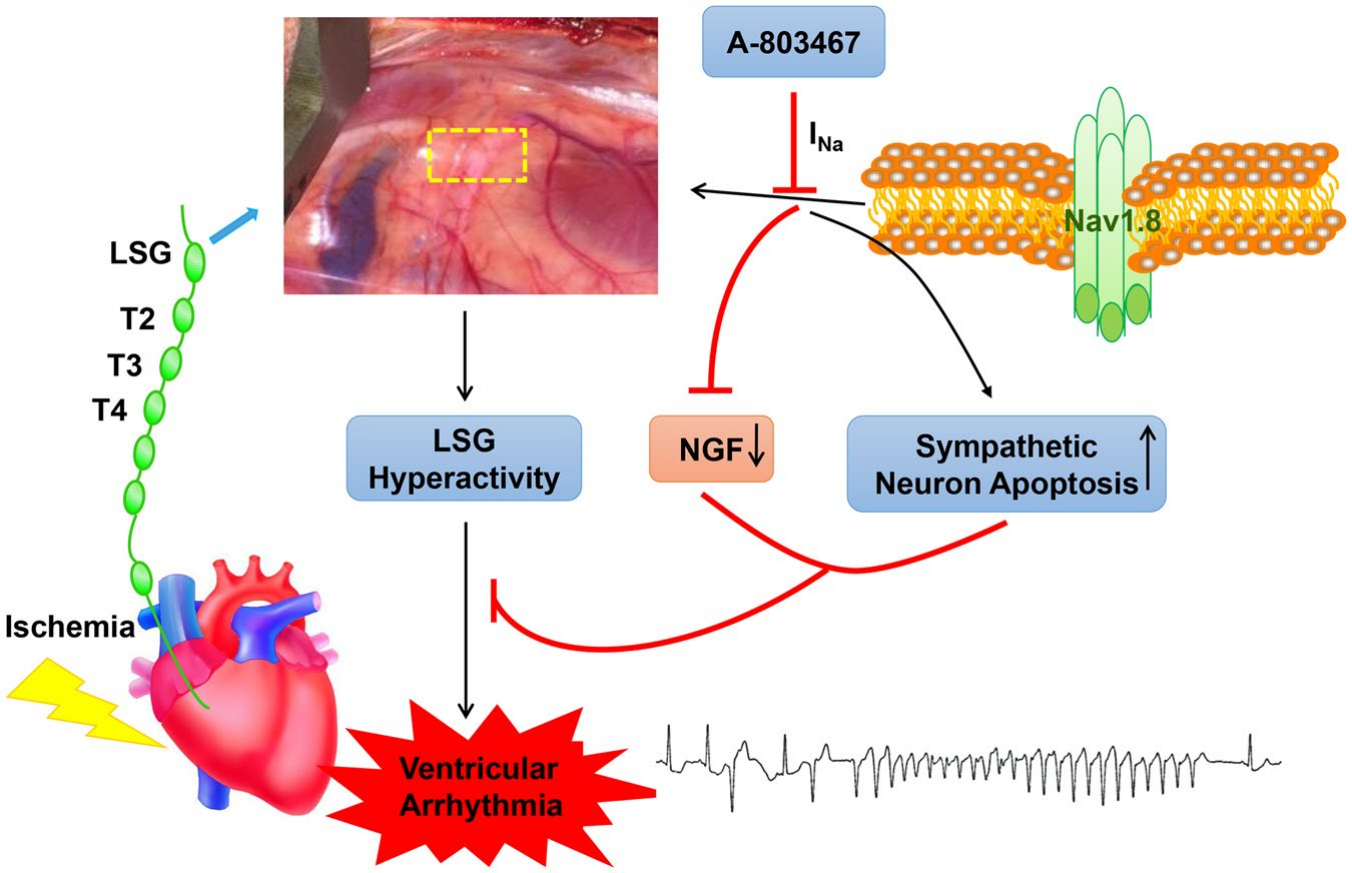
Schematic diagram depicting the potential role of Nav1.8 channel in LSG and VA. LSG hyperactivity contributes to the VA under acute ischemia stress. Whereas, blocking Nav1.8 channel with its specific blocker A-803467 could inhibit LSG activity through decreased NGF expression and increased sympathetic neuron apoptosis, which subsequently attenuates ischemia induced VA.

The present study demonstrated that blocking the Nav1.8 channel in the LSG with A-803467 prolongs the ventricular APD_80_ and ERP in a concentration-dependent manner in normal hearts. Furthermore, A-803467 administration into the LSG significantly attenuates VA in ischemic hearts. The decreased LSG activity, partly due to the decreased NGF expression and increased apoptosis of sympathetic neurons in the LSG, contributes to the protective function of the Nav1.8 channel blockade against VA (Fig. 7).

### LSG activity and the Nav1.8 channel

It is well known that the autonomic nervous system can modulate cardiac electrophysiology. Sympathetic stimulation increases the instability of cardiac electrophysiology, as demonstrated by the shortened ventricular APD and ERP, while sympathetic denervation or vagus nerve stimulation exerts the opposite effect^21–24^. The LSG is the key element in the cardiac autonomic nervous system. Studies from our team and other groups have confirmed that LSG inhibition, whether by direct LSG excision^8, 9, 25^, spinal cord stimulation according to the cardiac neuronal hierarchy theory^26^, or low-level vagus nerve stimulation^27^, can stabilize cardiac electrophysiology and suppress susceptibility to ischemia induced VA. In the present study, we first reported that blocking the Nav1.8 channel with A-803467 attenuates the activity of the LSG in a concentration-dependent manner, as demonstrated by the blunted SBP elevation in response to LSG electrical stimulation. In addition to LSG, right stellate ganglion (RSG) also plays important function in regulation ventricular electrophysiology and ischemic VA^2^. We assessed the effect of A-803467 on RSG function. Interestingly, decreased RSG function was found after the injection of A-803467, as demonstrated by the attenuated maximal sinus rate change at the same simulation voltage (see Supplementary Fig. S3). However, the deficiency is that the effects of A-803467 injection into RSG on ventricular electrophysiology and ischemic VA were not accessed. Whether A-803467 injection into the RSG plays antiarrhythmic effect similar with the effect in LSG remain unclear. Even though, our results provide direct evidence that the Nav1.8 channel could affect sympathetic activity and that this effect may contribute to the regulation of cardiac electrophysiological properties.

The function of the Nav1.8 channel in neuropathic pain has been well investigated^13^. In contrast to most other sodium channels, studies have demonstrated that the inactivation rate of Nav1.8 channel was slow. However, the recovery rate of Nav1.8 channel was quick^11, 28^. In addition, Nav1.8 channel was characterized by depolarized voltage dependence of inactivation^28^, which indicates that it is remain available for operation even when other sodium channels cannot respond to stimuli. Due to these unique properties, Nav1.8 channel contributes a substantial fraction of the transmembrane current that flows during the rising phase of the neuronal action potential^29^. Studies have demonstrated that the expression and electrophysiological function of the Nav1.8 channel are altered after direct spinal nerve ligation^12^. In addition, inhibition of the Nav1.8 channel, either by siRNA silencing or knock-down, can effectively alleviate pain symptoms in rodent pain models^30, 31^. A-803467, a small molecule that blocks the Nav1.8 channel 300- to 1000-fold more potently than other voltage-gated sodium channels, was recently discovered^15^. A-803467 attenuates neuropathic pain by inhibiting neuronal activity in a dose-dependent manner^16^. All these results indicate that the Nav1.8 channel plays important roles in alleviating pain through regulating the activity of afferent nerves. In the present study, we found that injection of a Nav1.8 channel blocker into the LSG prolonged ventricular APD_80_ and ERP in normal hearts and attenuated VA in ischemic hearts. These results further confirm that the Nav1.8 channel in the LSG contributes to the regulation of sympathetic activity and suggest a novel target for VA treatment.

### Possible mechanisms underlying the Nav1.8 channel blockade and decreased LSG activity

The cardiac sympathetic afferent reflex is an important contributor to the exaggerated sympathoexcitation in cardiovascular diseases, such as ischemia-induced heart failure and hypertension^32, 33^. Stimulating cardiac sympathetic afferent nerve endings caused an excitatory response of sympathetic nerve activity, while blocking cardiac sympathetic afferent inputs attenuated the sympathetic nerve activity^34, 35^. Studies have demonstrated that the LSG participates in the transition of cardiac sympathetic afferent signal from the heart to the spinal cord^36^. In addition, sympathetic afferent cell bodies have been identified in the LSG^37, 38^. Considering that the Nav1.8 channel is mainly expressed in primary afferent neurons^39^, the decreased sympathetic activity in the present study may be attributed to the attenuated cardiac sympathetic afferent reflex. However, the exact mechanism by which A-803467 regulates LSG afferent neuronal activity remains to be clarified.

Blasius A. L. *et al*. reported that mice carrying the Possum mutation in SCN10A, which enhances Nav1.8 channel activity and neuronal excitability, exhibit marked R-R variability upon scruffing^40^. Furthermore, these effects of the Possum mutation were attenuated by atropine infusion. Therefore, it is possible that Nav1.8 channel regulates the content of neurotransmitters and/or neuropeptides. Previous studies have shown that NGF promotes LSG remodeling and contributes to the increased ischemia-related VA^6, 20^. We detected mRNA expression of NGF in the LSG collected after ischemia, and the data indicated that A-803467 significantly attenuated the NGF expression. It has been demonstrated that the NGF levels in the LSG were not changed at 3.5 hours post-ischemia^41^. Our results also confirmed that the mRNA expression of NGF after 60 min ischemia was similar with that in normal canines (see Supplementary Fig. S2). Thus, the decreased NGF expression may be responsible for the attenuated LSG activity induced by A-803467. More importantly, it has been validated that NGF protects neuron cells against apoptosis^42, 43^. In the absence of NGF, sympathetic neurons die by apoptosis in a transcription-dependent manner^43^. The decreased NGF expression in the present study may lead to neuron apoptosis in the LSG. However, further research is warranted to clarify the detailed mechanisms by which the Nav1.8 channel regulates NGF expression. In addition, other mechanisms for the Nav1.8 channel regulation of NGF expression and neuron apoptosis may exist.

### Study limitations

The present study has several limitations. First, the present study was done under pentobarbital anesthesia, which may affect the autonomic nervous system. However, the animals were anesthetized similarly, and the ischemia was induced by the same method. The only difference between the various groups was the treatment: DMSO or different concentrations of A-803467. Thus, the effect of anesthesia on the LSG activity could be counteracted among the different groups. Second, the protein expression of Nav1.8 in the canine LSG under physiological and pathophysiological conditions such as acute ischemia was not examined because of the lack of a dog-specific antibody against Nav1.8. However, mRNA expression of Nav1.8 was found in the LSG (see Supplementary Fig. S4). Third, increased myocardial dispersion of repolarization was associated with the pro-arrhythmia effect of sympathetic hyperactivity^44, 45^. Despite the APD and ERP, the assessment of the dispersion of repolarization, directly evaluated with a 56-electrode sock, could further elucidate the potential role of the Nav1.8 channel in the stellate ganglion in cardiac electrophysiology. Fourth, in addition to the LSG function, direct neural activity recording may be better to reflect the LSG activity. Last, the long-term effect of Nav1.8 channel blockade on cardiac electrophysiology was not examined. However, apoptosis of sympathetic neurons was found in the LSG. It is speculated that the effect of Nav1.8 channel blockade lasts a long time.

### Clinical perspectives

Clinical and basic studies demonstrate that an activated cardiac sympathetic nervous system, especially the LSG, plays a central role in the initialization and maintenance of VA. Left cardiac sympathetic denervation (LCSD) has been clinically applied to treat patients with refractory VA or electrical storm^8, 9^, which pose high risks for VF and sudden cardiac death. LCSD has usually been achieved through direct surgical resection or resection through video-assisted thoracic surgery. However, the clinical use of sympathetic denervation is limited due to undesirable complications such as Horner’s syndrome and accidental hemorrhages. Our study suggests that injection of the Nav1.8 channel-specific blocker A-803467 (minimum effective dose: 15 mM in 0.1 ml) into the LSG attenuates VA severity in an ischemic model, possibly by inhibiting the LSG activity via attenuating NGF expression and increasing sympathetic neuron apoptosis. Injection of a Nav1.8 channel-specific blocker into the LSG under thoracoscope support may avoid the main side effects of conventional surgical LSG excision. Further research is warranted to evaluate the safety and efficiency of this new method.

### Conclusion

In the present study, we found that microinjection of a specific Nav1.8 channel inhibitor into the LSG increases the cardiac electrophysiology stability and attenuates VA induced by acute ischemia. The decreased LSG activity, partly due to the decreased NGF and c-fos expression and increased sympathetic neuron apoptosis in the LSG, contributes to the protective function of the Nav1.8 channel blockade against VA. Targeting the Nav1.8 channel in the LSG may be a novel approach to attenuate ischemia-induced VA.

## Materials and Methods

### Experimental animals and surgical preparation

All animal experiments were performed according to the National Institutes of Health guidelines and were approved by the Animal Care and Use Committees of Renmin Hospital of Wuhan University. Male canines with body weights of 18~20 kg were anesthetized with 3% sodium pentobarbital with an initial dose of 1 ml/kg and a maintenance dose of 2 ml/h. The ECG signals were recorded using a computer-based Lab System (Lead 7000, Jinjiang Inc., Chengdu, China) with filters between 0.5 and 30 Hz. The sampling rate of all recorded signals was 4 KHz. A heating pad was used to maintain the core body temperature of canines at 36.5 °C ± 1.5 °C.

A bilateral thoracotomy was performed at the fourth intercostal space. Acute ischemia was established by occlusion of the LAD for 1 h as described in our previous study^21^ and confirmed by the development of acute ST-segment and T-wave changes on surface ECG. The VA was recorded by the Lead 7000 system and then numbered manually after the experiment. The VA was defined according to the Lambeth Conventions^46^. The VPB was defined as one premature ventricular beat. The salvo beat was defined as two consecutive premature ventricular beats. VT was defined as three or more consecutive premature ventricular beats. VF was defined as tachycardia with random electrocardiogram morphology associated with the loss of arterial BP that degenerated into ventricular asystole.

### Administration of the Nav1.8 blocker A-803467

Forty canines were randomly divided into a DMSO group, a low concentration A-803467 group (10 mM), a moderate concentration A-803467 group (15 mM), and a high concentration A-803467 group (20 mM). A final volume of 0.1 ml of A-803467 or DMSO was injected into the LSG at four points under direct visual DMSO to ensure optimal injection. A-803467 (Sigma Aldrich, A3109, St. Louis, Missouri, USA) was dissolved in DMSO, stored at a concentration of 50 mM, and diluted to its final concentration before use.

### Measurement of LSG function

The LSG was separated and identified as previously described^47^. The relative change of maximal SBP in response to direct electrical stimulation of the LSG reflected the LSG function^26^. LSG function was detected at baseline and 30 min after the administration of A-803467. A Grass-S88 stimulator (Astro-Med, West Warwick, Rhode Island, USA) was used to apply high-frequency stimulation (20 Hz, 0.1 ms pulse duration, and voltages of 20 V, 40 V, 60 V, and 80 V) to the LSG. Subsequently, a voltage/BP response curve was constructed as described in our previous studies^47^. The high-frequency stimulation lasted 30 seconds, and the next measurement was not recorded until the BP returned to the baseline level.

### Electrophysiological measurements

The S1–S2 method, which comprised eight basic stimulation (S1–S1 = 330 ms) at twice the diastolic threshold and one premature stimulation (S2), was used to examine the ventricular ERP at three epicardial sites: LVA, LVB and LVM. The ERP was defined as the longest S1–S2 interval that failed to capture the ventricles^47^. The S1–S2 interval was started at 250 ms and decreased initially by 10 ms and then 2 ms until refractoriness was achieved (S1:S2 = 8:1).

Left ventricle monophasic APD was recorded at the LVA, LVM, and LVB with a custom-made Ag-AgCl electrode. Paired platinum stimulating electrodes were positioned on the epicardial surface of the right atrial appendage (RAA) with regular pacing (pacing cycle length = 330 ms). Monophasic APD signals were filtered at 0.4 to 1200 Hz and analyzed using the Lead 7000B Workstation (Jinjiang Inc.). APD measured at 80% repolarization was defined as the APD_80_.

### Polymerase Chain Reaction (PCR) and TUNEL staining

At the end of the experiment, the LSG was excised and washed with saline. The tissue for PCR was dissected into small portions, snap-frozen in liquid nitrogen, and then maintained at −80 °C until use. For TUNEL staining, the tissue was fixed by 4% paraformaldehyde solution and embedded in paraffin. The paraffin-embedded LSG was cut transversely into 5-μm sections.

Total RNA was extracted from LSG tissue with the TRIzol reagent (Roche, Basle, Switzerland Indiana) according to the manufacturer’s instructions, and the concentration was measured with a NanorDrop 2000. A total of 2 μg RNA was used to synthesize cDNA with the Transcriptor First Stand cDNA Synthesis Kit (Roche). Quantitative real-time PCR (qRT-PCR) was performed with the LightCycler 480 SYBR Green I Master Mix (Roche) using the LightCycler 480 real-time PCR system (Roche) according to the manufacturer’s instructions. Primer pairs were shown in Online Supplemental Table 1. The PCR conditions were as follows: initial denaturation at 95 °C for 10 min, followed by 40 cycles of 95 °C for 10 seconds (denaturation), 60 °C for 10 seconds (annealing), and 72 °C for 20 seconds (extension). The relative expression levels of mRNA were normalized to the reference gene GAPDH. All reactions were conducted in triplicate, and the data were calculated using the 2^−ΔΔCT^ method.

TUNEL assay was performed using the ApopTag Plus *In Situ* Apoptosis Fluorescein Detection Kit (Millipore, Boston, Massachusetts, USA) according to the manufacturer’s protocol. Double staining of TH (Abcam, Cambridge, England) and TUNEL was applied to evaluate sympathetic neuron apoptosis. The nuclei were stained with 4,6-diamidino-2-phenylindole (DAPI). Images were all obtained with a fluorescence microscope (Olympus Dx51) and DP2-BSW software (Version 2.2), and the images were analyzed with Image-Pro Plus (Version 6.0) in a blinded manner.

### Statistical analysis

All variables were expressed as means ± standard deviation (SD) and were analyzed by one-way ANOVA. To compare the incidence of VF between groups, Fisher’s exact test was used. The SPSS PASW Statistics (version 18.0) and GraphPad Prism software (version 5.0) were used for data analysis and graphing. The significance level was set at p < 0.05.

## Electronic supplementary material

SI without changes marked

## References

[1] Shen MJ, Zipes DP (2014). Role of the autonomic nervous system in modulating cardiac arrhythmias. Circ. Res..

[2] Puddu PE (1988). Prevention of postischemic ventricular fibrillation late after right or left stellate ganglionectomy in dogs. Circulation.

[3] Schwartz PJ, Stone HL, Brown AM (1976). Effects of unilateral stellate ganglion blockade on the arrhythmias associated with coronary occlusion. Am. Heart J..

[4] Zhou S (2008). Spontaneous stellate ganglion nerve activity and ventricular arrhythmia in a canine model of sudden death. Heart Rhythm..

[5] Swissa M (2004). Long-term subthreshold electrical stimulation of the left stellate ganglion and a canine model of sudden cardiac death. J. Am. Coll. Cardiol..

[6] Cao JM (2000). Nerve sprouting and sudden cardiac death. Circ. Res..

[7] Schwartz PJ, Stone HL (1980). Left stellectomy in the prevention of ventricular fibrillation caused by acute myocardial ischemia in conscious dogs with anterior myocardial infarction. Circulation.

[8] Vaseghi M (2014). Cardiac sympathetic denervation in patients with refractory ventricular arrhythmias or electrical storm: intermediate and long-term follow-up. Heart Rhythm..

[9] Bourke T (2010). Neuraxial modulation for refractory ventricular arrhythmias: value of thoracic epidural anesthesia and surgical left cardiac sympathetic denervation. Circulation.

[10] Odero A, Bozzani A, De Ferrari GM, Schwartz PJ (2010). Left cardiac sympathetic denervation for the prevention of life-threatening arrhythmias: the surgical supraclavicular approach to cervicothoracic sympathectomy. Heart Rhythm..

[11] Sangameswaran L (1996). Structure and function of a novel voltage-gated, tetrodotoxin-resistant sodium channel specific to sensory neurons. J. Biol. Chem..

[12] Gold MS (2003). Redistribution of Na(V)1.8 in uninjured axons enables neuropathic pain. J. Neurosci..

[13] Han C, Huang J, Waxman SG (2016). Sodium channel Nav1.8: Emerging links to human disease. Neurology.

[14] Daou, I. *et al*. Optogenetic Silencing of Nav1.8-Positive Afferents Alleviates Inflammatory and Neuropathic Pain. *eNeuro*. 3, pii: ENEURO.0140-15.2016, doi:10.1523/ENEURO.0140-15 (2016). 10.1523/ENEURO.0140-15.2016 PMC4794527 27022626

[15] Jarvis MF (2007). A-803467, a potent and selective Nav1.8 sodium channel blocker, attenuates neuropathic and inflammatory pain in the rat. Proc. Natl. Acad. Sci. USA.

[16] McGaraughty S (2008). A selective Nav1.8 sodium channel blocker, A-803467 [5-(4-chlorophenyl-N-(3,5-dimethoxyphenyl)furan-2-carboxamide], attenuates spinal neuronal activity in neuropathic rats. J. Pharmacol. Exp. Ther..

[17] Qi B (2014). Nav1.8 channels in ganglionated plexi modulate atrial fibrillation inducibility. Cardiovasc. Res..

[18] Chen, X. *et al*. Neuronal Nav1.8 Channels as a Novel Therapeutic Target of Acute Atrial Fibrillation Prevention. *J. Am. Heart Assoc*. 5 (2016). 10.1161/JAHA.116.004050 PMC5210368 27806967

[19] Sagar SM, Sharp FR, Curran T (1988). Expression of c-fos protein in brain: metabolic mapping at the cellular level. Science.

[20] Chen PS (2001). Sympathetic nerve sprouting, electrical remodeling and the mechanisms of sudden cardiac death. Cardiovasc. Res.

[21] Huang B (2014). Renal sympathetic denervation modulates ventricular electrophysiology and has a protective effect on ischaemia-induced ventricular arrhythmia. Exp. Physiol..

[22] Gu Y (2012). Assessment of ventricular electrophysiological characteristics at periinfarct zone of postmyocardial infarction in rabbits following stellate ganglion block. J. Cardiovasc. Electrophysiol.

[23] Ng GA (2009). Sympathetic nerve stimulation produces spatial heterogeneities of action potential restitution. Heart Rhythm..

[24] Ng GA, Brack KE, Patel VH, Coote JH (2007). Autonomic modulation of electrical restitution, alternans and ventricular fibrillation initiation in the isolated heart. Cardiovasc. Res..

[25] Schwartz PJ (2014). Cardiac sympathetic denervation to prevent life-threatening arrhythmias. Nat. Rev. Cardiol.

[26] Wang S (2015). Spinal cord stimulation protects against ventricular arrhythmias by suppressing left stellate ganglion neural activity in an acute myocardial infarction canine model. Heart Rhythm..

[27] Yu L (2016). Chronic Intermittent Low-Level Stimulation of Tragus Reduces Cardiac Autonomic Remodeling and Ventricular Arrhythmia Inducibility in a Post-Infarction Canine Model. J. Am. Coll. Cardiol. EP.

[28] Akopian AN, Sivilotti L, Wood JN (1996). A tetrodotoxin-resistant voltage-gated sodium channel expressed by sensory neurons. Nature.

[29] Renganathan M, Cummins TR, Waxman SG (2001). Contribution of Na(v)1.8 sodium channels to action potential electrogenesis in DRG neurons. J. Neurophysiol..

[30] Joshi SK (2006). Involvement of the TTX-resistant sodium channel Nav 1.8 in inflammatory and neuropathic, but not post-operative, pain states. Pain.

[31] Gaida W, Klinder K, Arndt K, Weiser T (2005). Ambroxol, a Nav1.8-preferring Na(+) channel blocker, effectively suppresses pain symptoms in animal models of chronic, neuropathic and inflammatory pain. Neuropharmacology.

[32] Malliani A, Montano N (2002). Emerging excitatory role of cardiovascular sympathetic afferents in pathophysiological conditions. Hypertension.

[33] Minisi AJ, Thames MD (1991). Activation of cardiac sympathetic afferents during coronary occlusion. Evidence for reflex activation of sympathetic nervous system during transmural myocardial ischemia in the dog. Circulation.

[34] Wang W, Ma R (2000). Cardiac sympathetic afferent reflexes in heart failure. Heart Fail. Rev..

[35] Wang HJ, Wang W, Cornish KG, Rozanski GJ, Zucker IH (2014). Cardiac sympathetic afferent denervation attenuates cardiac remodeling and improves cardiovascular dysfunction in rats with heart failure. Hypertension.

[36] Hopkins DA, Armour JA (1989). Ganglionic distribution of afferent neurons innervating the canine heart and cardiopulmonary nerves. J. Auton. Nerv. Syst..

[37] Armour JA (1986). Activity of *in situ* stellate ganglion neurons of dogs recorded extracellularly. Can J Physiol Pharmacol.

[38] Bosnjak ZJ, Kampine JP (1989). Cardiac sympathetic afferent cell bodies are located in the peripheral nervous system of the cat. Circ. Res..

[39] Djouhri L (2003). The TTX-resistant sodium channel Nav1.8 (SNS/PN3): expression and correlation with membrane properties in rat nociceptive primary afferent neurons. J. Physiol.

[40] Blasius AL (2011). Hypermorphic mutation of the voltage-gated sodium channel encoding gene Scn10a causes a dramatic stimulus-dependent neurobehavioral phenotype. Proc. Natl. Acad. Sci. USA.

[41] Zhou S (2004). Mechanisms of cardiac nerve sprouting after myocardial infarction in dogs. Circ. Res..

[42] Kristiansen M, Ham J (2014). Programmed cell death during neuronal development: the sympathetic neuron model. Cell Death Differ.

[43] Deshmukh M, Johnson EJ (1997). Programmed cell death in neurons: focus on the pathway of nerve growth factor deprivation-induced death of sympathetic neurons. Mol. Pharmacol..

[44] Yagishita D (2015). Sympathetic nerve stimulation, not circulating norepinephrine, modulates T-peak to T-end interval by increasing global dispersion of repolarization. Circ. Arrhythm. Electrophysiol.

[45] Vaseghi M (2013). Modulation of regional dispersion of repolarization and T-peak to T-end interval by the right and left stellate ganglia. Am. J. Physiol. Heart Circ. Physiol.

[46] Walker MJ (1988). The Lambeth Conventions: guidelines for the study of arrhythmias in ischaemia infarction, and reperfusion. Cardiovasc. Res..

[47] Huang B (2014). Left renal nerves stimulation facilitates ischemia-induced ventricular arrhythmia by increasing nerve activity of left stellate ganglion. J. Cardiovasc. Electrophysiol..

