# Supplementary material for: Blocking the Nav1.8 channel in the left stellate ganglion suppresses ventricular arrhythmia induced by acute ischemia in a canine model
Source: Sci Rep. 2017 Apr 3;7:534. doi: 10.1038/s41598-017-00642-6 (PMC5428783; doi:10.1038/s41598-017-00642-6)
Supplement: Supplementary file 1 — SI without changes marked [file 41598_2017_642_MOESM1_ESM.doc]

**Blocking the Nav1.8 channel in the left stellate ganglion suppresses ventricular arrhythmia induced by acute ischemia in a canine model**

**Short title: Nav1.8 channel and ventricular arrhythmia**

Lilei Yu, MD, PhD^1^*,, Menglong Wang, MD^1^,*, Dan Hu, MD, PhD^1,2^, Bing Huang, MD, PhD^1^, Liping Zhou, MD^1^, Xiaoya Zhou, MD, PhD^1^, Zhuo Wang, MD, PhD^1^, Songyun Wang, MD, PhD^1^, Hong Jiang, MD^1,#^

^1^ Department of Cardiology, Renmin Hospital of Wuhan University; Cardiovascular Research Institute, Wuhan University; Hubei Key Laboratory of Cardiology, Wuhan, China

^2^ Masonic Medical Research Laboratory, 2150 Bleecker Street, Utica, New York, 13501-1787, USA.

***These authors contributed equally to this work as co-first authors.**

**^#^ Reprint requests and correspondence:**

Professor Hong Jiang,

Department of Cardiology, Renmin Hospital of Wuhan University,

238 Jiefang Road, Wuhan 430060, China.

Email: whujianghong@163.com

Tel: 86-27-88041911

Fax: 86-27-88042293

**Supplementary Information**

**The original traces for APD and ERP**

As shown in Supplementary Fig. S1, the APD markedly increased in the 20 mM A-803467 treatment group. The APD was 210 ms (Baseline 206 ms) and 268 ms (Baseline 212 ms) at 330 ms pacing cycle length in the DMSO group and 20 mM A-803467 treatment group. Besides, the ERP was prolonged by A-803467 treatment. As the figure showed, the ERP was 158 ms (Baseline 156 ms) and 178 ms (Baseline 160 ms) in the DMSO group and 20 mM A-803467 treatment group, respectively.

**The mRNA expression of NGF in the LSG collected from the DMSO group and normal canines**

To explore the effect of 60 min ischemia on the expression of NGF in the LSG, LSG tissues from the DMSO group and 3 normal canines without any intervention were collected and the mRNA expression of NGF were evaluated by qRT-PCR. The results showed that the expression of NGF in the DMSO group was similar to that in the normal canines (Supplementary Fig. S2).

**A-803467 injection decreased RSG function**

RSG function was defined as the maximal heart rate change in response to RSG electrical stimulation (20 Hz, 0.1 ms duration at different voltages). To explore the potential effect of Nav1.8 channel blocker A-803467 on RSG function, another 40 canines were included and the DMSO and same concentrations of A-803467 were injected into RSG and the RSG function was detected 30 min after the injection. As shown in Supplementary Fig. S3, decreased RSG activity was found after the injection of A-803467, as demonstrated by the attenuated maximal sinus rate change at the same simulation voltage.

**Identification of the existence of the Nav1.8 channel in the LSG**

The cDNA of three canines from control group was used to verify the Nav1.8 mRNA expression in the LSG. Reverse transcriptase PCR was applied for gene amplification. Then the PCR product was sampled into 3% agarose gel electrophoresis with 6× loading buffer. The band was identified using the gel imaging system (Tanon-1600R, Tanon Science & Technology Co., Ltd. Shanghai, China). The primer pare for Nav1.8 was shown in Supplementary Table S1.

As shown in Supplementary Fig. S4, the reference band GAPDH was around 200 bp, while the band for Nav1.8 was around 300 bp. This result validated the expression of Nav1.8 channel in the LSG.

**Supplementary Figure legends:**

**Supplementary Fig. S1.** The original traces for APD (A) and ERP (B) were shown. BS = Baseline.

**Supplementary Fig. S2.** The mRNA expression in the LSG from the DMSO group and the normal canines. n.s. = no significance.

**Supplementary Fig. S3.** Blocking Nav1.8 channel in the RSG with A-803467 decreased RSG function. RSG = Right stellate ganglion. *p＜.05 vs DMSO group, ^#^ p＜.05 vs 10mM group.

**Supplementary Fig. S4.** The mRNA expression of Nav1.8 channel in the LSG.

**Supplementary Fig. S1**


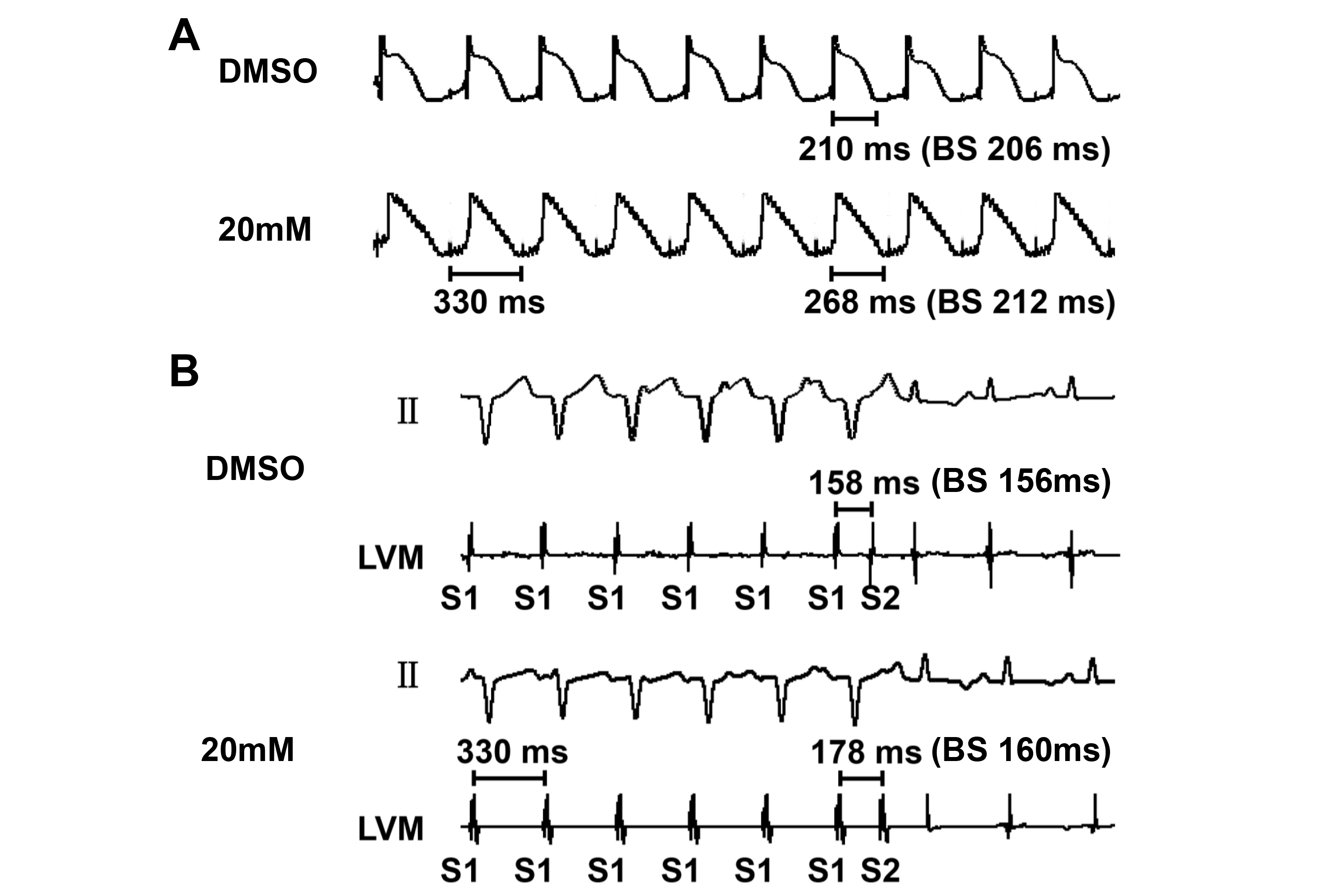


**Supplementary Fig. S2**

**
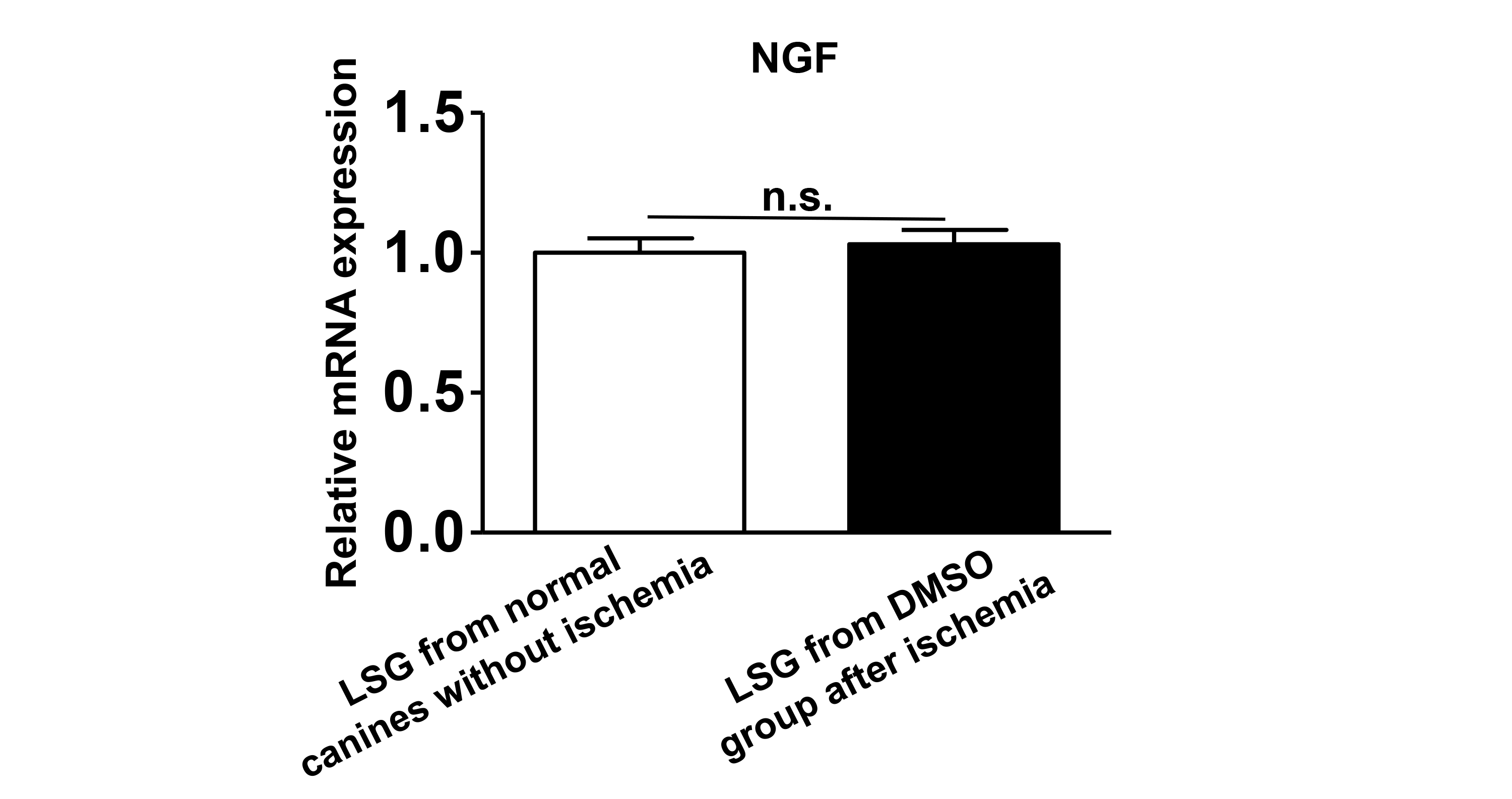
**

**Supplementary Fig. S3**


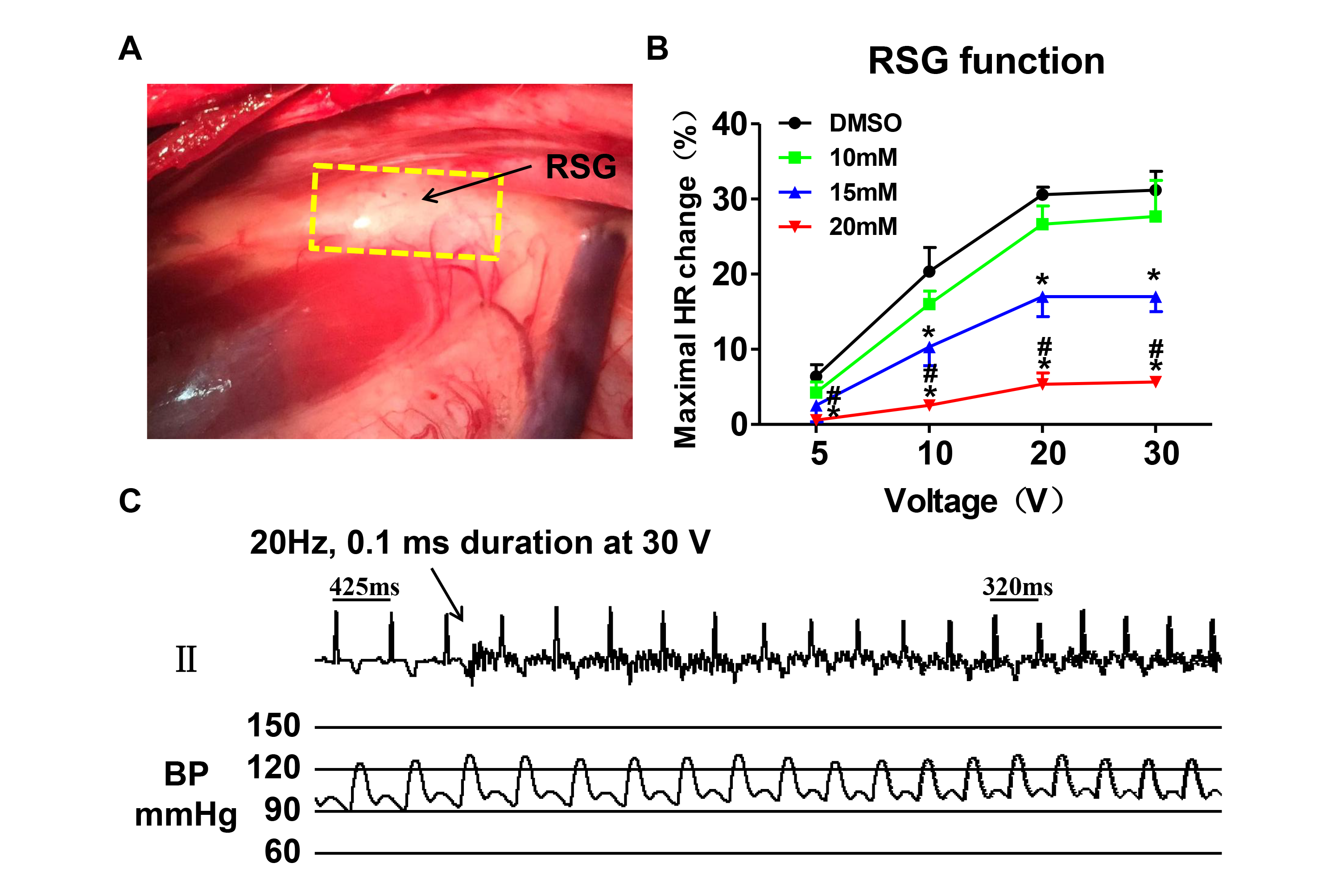


**Supplementary Fig. S4**


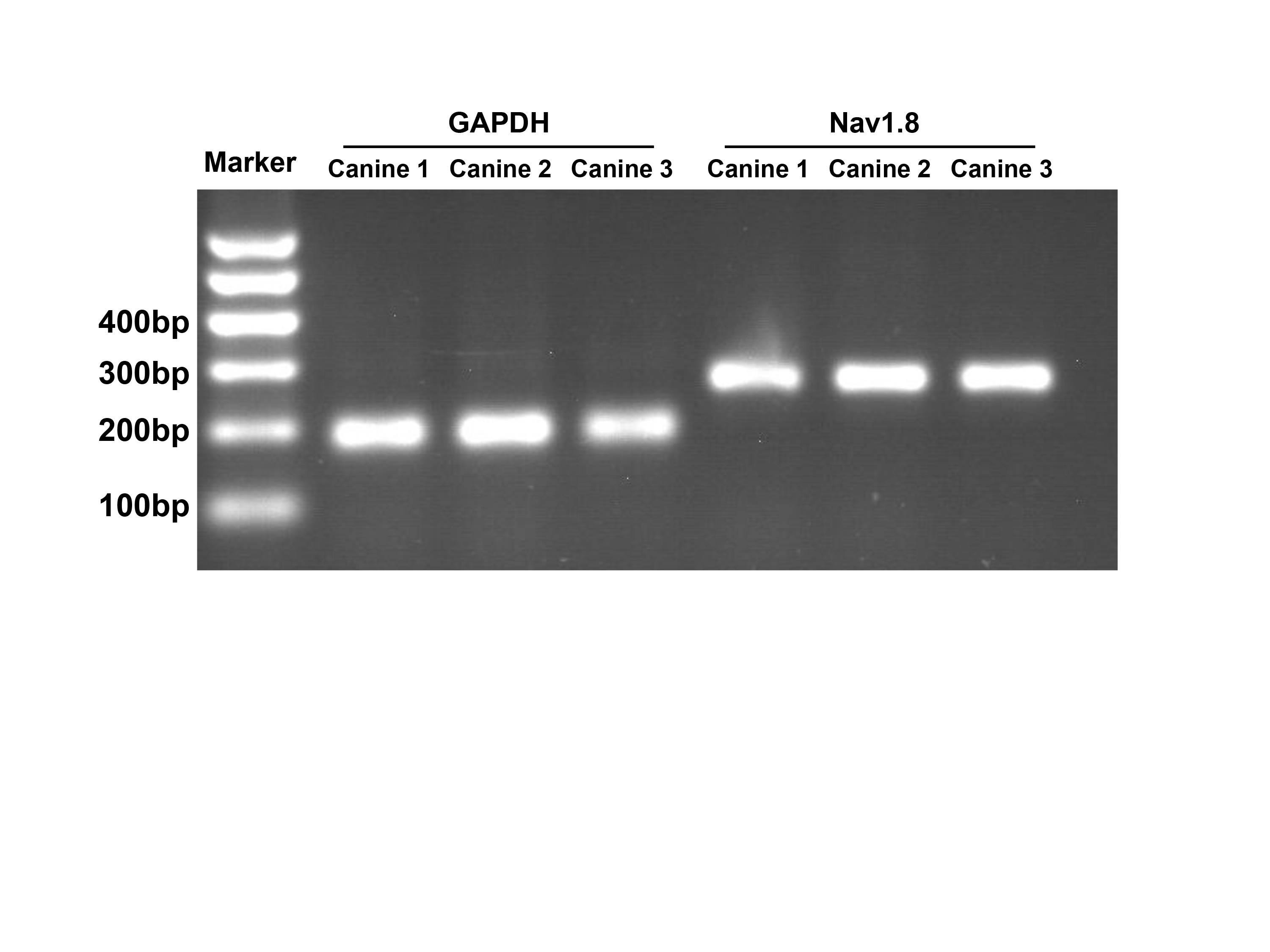


**Supplementary Table S1. The primer pares used in the present study**

| Gene name | Primer sequence | Amplion size, bp |
| --- | --- | --- |
| GAPDH | F: TGATGCTGGTGCTGAGTATGTT | 183 |
|  | R: TTGCTGACAATCTTGAGGGAGT |  |
| NGF | F: ACAGGAGCAAGCGGTCTTCG | 265 |
|  | R: TGGGTGGTGGTGCAGTAGGA |  |
| c-fos | F: CAGTGCCAACTTCATCCCG | 285 |
|  | R: GCAGCCATCTTATTCCTTTCC |  |
| Nav1.8 | F: AGCCAGCAAAGAAGAATGAGAAGA | 272 |
|  | R:CCTGCTTGGCAGCAATTCTCT |  |

F: Forward sequences; R: Reverse sequences
